# Variante da Subunidade Beta 3 da Proteína G ( *GNB3* ) Está Associada a Alterações Bioquímicas em Pacientes Brasileiros com Hipertensão

**DOI:** 10.36660/abc.20230396

**Published:** 2023-12-07

**Authors:** Lívia da Cunha Agostini, Nayara Nascimento Toledo Silva,, Ana Cláudia Faria Lopes, André Sacramento Melo, Luciana Soares Moreira Bicalho, Tamires Cunha Almeida, Vanessa de Almeida Belo, Wendel Coura-Vital, Luiz Fernando de Medeiros Teixeira, Angélica Alves Lima, Glenda Nicioli da Silva

**Affiliations:** 1 Universidade Federal de Ouro Preto Ouro Preto MG Brasil Universidade Federal de Ouro Preto (UFOP), Ouro Preto , MG – Brasil; 2 Universidade Federal de Ouro Preto Departamento de Análises Clínicas Ouro Preto MG Brasil Universidade Federal de Ouro Preto (UFOP) – Departamento de Análises Clínicas (DEACL), Ouro Preto , MG – Brasil; 3 Butantan Institute São Paulo SP Brasil Butantan Institute , São Paulo , SP – Brasil; 4 Universidade Federal de Ouro Preto CiPharma DEFAR Ouro Preto MG Brasil Universidade Federal de Ouro Preto – Programa de Pós-Graduação em Ciências Farmacêuticas ( CiPharma ) – Departamento de Farmácia ( DEFAR ), Ouro Preto , MG – Brasil; 5 Universidade Federal de Ouro Preto CiPharma DEACL Ouro Preto MG Brasil Universidade Federal de Ouro Preto – Programa de Pós-Graduação em Ciências Farmacêuticas ( CiPharma ) – Departamento de Análises Clínicas ( DEACL ), Ouro Preto , MG – Brasil

**Keywords:** Alelos, Reações Bioquímicas, Genótipo, Hipertensão

## Abstract

**Fundamento:**

Genes e suas variantes associadas a fatores ambientais contribuem para o desenvolvimento do fenótipo hipertenso. O gene da subunidade beta 3 da proteína G ( *GNB3* ) está envolvido no processo de sinalização intracelular e suas variantes têm sido relacionadas à suscetibilidade à hipertensão arterial.

**Objetivo:**

Determinar a associação da variante *GNB3* (rs5443:C>T) com a hipertensão arterial, parâmetros bioquímicos, idade e obesidade em indivíduos hipertensos e normotensos de Ouro Preto, Minas Gerais.

**Método:**

A identificação das variantes foi realizada por PCR em tempo real, utilizando o sistema TaqMan®, em amostras de 310 pacientes (155 hipertensos e 155 normotensos). Análises bioquímicas (função renal, perfil lipídico e glicemia) foram realizadas a partir do soro por meio de espectrofotometria UV/Vis e eletrodo íon-seletivo. Foi utilizado um modelo de regressão logística múltipla para identificar fatores associados à hipertensão arterial. A análise das variáveis contínuas com distribuição normal foi realizada usando o teste t de Student não pareado; dados não normais foram analisados usando o teste de Mann-Whitney. Valores de p < 0,05 foram considerados significativos.

**Resultados:**

A variante rs5443:C>T não esteve associada à hipertensão arterial na população avaliada (p = 0,88). Em relação às medidas bioquímicas, o alelo T esteve associado a níveis elevados de triglicerídeos, glicose e ácido úrico em indivíduos hipertensos (p < 0,05).

**Conclusão:**

Os presentes resultados mostram a importância do diagnóstico genético para prevenir as causas e consequências de doenças e sugerem que a variante GNB3 rs5443:C>T pode estar associada a alterações no perfil bioquímico em indivíduos hipertensos.

## Graphical abstract

**Figura Central:**
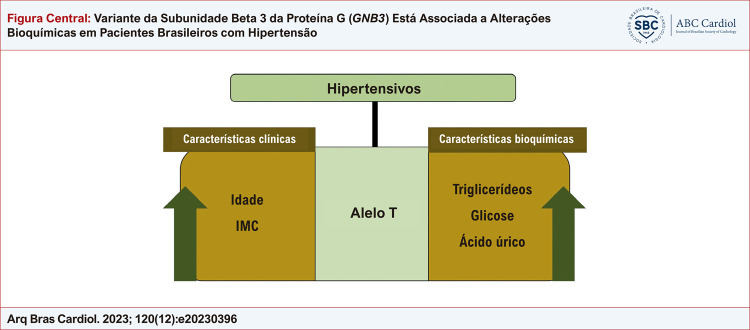
: Variante da Subunidade Beta 3 da Proteína G ( *GNB3* ) Está Associada a Alterações Bioquímicas em Pacientes Brasileiros com Hipertensão

## Introdução

A hipertensão arterial é uma doença crônica responsável por diversas doenças frequentemente associadas a distúrbios metabólicos. Afeta vários órgãos-alvo e pode levar a morte súbita, acidente vascular cerebral, doença arterial periférica, insuficiência cardíaca, infarto agudo do miocárdio e doença renal crônica. ^1 , 2^

A regulação fisiológica da pressão arterial e as alterações fisiopatológicas que levam à hipertensão arterial têm um componente genético. Pressupõe-se que 30% a 50% da variabilidade interindividual da pressão arterial pode ser estipulada geneticamente. ^3 , 4^ Foi relatado que a variante *GNB3* rs5443:C>T, localizada no cromossomo 12p13, na região do éxon 10, está associada com a hipertensão arterial. ^3 , 5^

As proteínas G fazem parte de uma superfamília de proteínas e estão inicialmente em estado inativo ligadas a receptores intracelulares. Quando ativadas, acionam enzimas amplificadoras e estimulam canais iônicos, realizando transdução de sinal. ^6^ A variante *GNB3* rs5443:C>T é responsável pela troca do alelo C por T, gerando *splicing* alternativo do éxon 9, eliminando 41 aminoácidos (498-620) da proteína, gerando a variante funcional truncada G3-s que exacerba a proteína G. ^6 , 7^ Isso desencadeia a sinalização intracelular que regula a disponibilidade de sódio e potássio. No estado hiperativo, a proteína G aumenta a retenção de sódio e água, contribuindo para o desenvolvimento da hipertensão arterial. ^7^

Alguns estudos investigaram a associação entre a variante *GNB3* rs5443:C>T e a pressão arterial em outras populações, mas os resultados são controversos. ^5 - 8^ No entanto, Chen et al. ^4^ discutiram como a variante *GNB3* rs5443:C>T pode servir como marcador genético precoce da sensibilidade da pressão arterial ao sal. A presença do polimorfismo gera uma proteína funcional que desencadeia a lipólise através das catecolaminas, alterando o perfil lipídico na corrente sanguínea. ^9^ Além disso, provoca uma redução da sensibilidade à insulina no tecido muscular e intensa reabsorção de sódio, favorecendo a hipertensão arterial. ^10^ Devido ao comprometimento endotelial/renal causado pela hipertensão arterial, há uma deficiência na excreção de algumas substâncias, como ureia, creatinina e ácido úrico, aumentando suas concentrações plasmáticas. ^1 , 9^

Levando em consideração a população global, a frequência do alelo C é de 67% e a do alelo T é de 33%. Grupos étnicos como europeus, africanos, afro-americanos, asiáticos e latino-americanos têm frequências dos alelos C e T em torno de 69% e 31%; 28% e 72%; 28% e 72%; 46% e 54%; 54% e 46%, respectivamente. ^11^ Estudos têm mostrado diferentes frequências em populações brasileiras diferentes. ^12 , 13^

Considerando a importância da variabilidade genética na hipertensão arterial, o presente estudo teve como objetivo determinar se a variante *GNB3* rs5443:C>T estava associada à hipertensão arterial e se influenciava a função renal, o perfil lipídico e a glicemia em uma amostra de pacientes brasileiros hipertensos e normotensos.

## Métodos

### Declaração ética

O presente estudo foi realizado de acordo com os critérios adotados pelo Comitê de Ética Universitária (CAAE 22455119.0.0000.5150), conforme resolução 466/2012.

### Desenho do estudo

O presente estudo caso-controle foi realizado em 2021 na cidade de Ouro Preto, Minas Gerais. Foram convidados a participar do estudo indivíduos presentes no Laboratório de Análises Clínicas da Faculdade de Farmácia da Universidade Federal de Ouro Preto para realização de exames bioquímicos. Aos que aceitaram, foi aplicado um questionário no aplicativo de smartphone KoBoToolbox, a fim de obter informações sobre dados sociodemográficos, comportamentais e de histórico médico. As medidas antropométricas como peso, altura e circunferência da cintura foram obtidas por meio de balança de bioimpedância, estadiômetro e fita métrica, respectivamente. Subsequentemente, foram coletadas amostras de sangue para avaliação bioquímica e molecular.

Após análise do questionário/prontuário, os indivíduos foram separados em dois grupos. Foram classificados como hipertensos aqueles que faziam uso de medicamentos para hipertensão e tinham diagnóstico prévio da doença no prontuário. Indivíduos que não faziam uso de medicação anti-hipertensiva e não possuíam diagnóstico de hipertensão no prontuário foram classificados como controles (normotensos).

O número da amostra foi definido para atingir o nível de significância de 95% que é crucial para estudos genéticos. Dessa maneira, o tamanho da amostra foi definido usando o programa OpenEpi, versão 3.01, com nível de confiança bilateral (1-alfa) de 95, poder de 80%, proporção de controles para casos de 1, proporção hipotética de controles com exposição de 33% de 8 e odds ratio de 2. Com isso, o tamanho da amostra foi estimado em aproximadamente 138 pacientes para os grupos controle e caso, totalizando 276 pacientes, segundo o teste de Kelsey. Ao final, o grupo de hipertensos incluiu 155 pacientes, sendo 87 mulheres e 68 homens com média de idade de 60,7 anos, e o grupo controle também incluiu 155 pacientes, 85 mulheres e 70 homens com média de idade de 58,2 anos.

### Dosagens bioquímicas

Para as análises bioquímicas, os participantes jejuaram por 8 horas. Os perfis lipídicos (triglicerídeos, colesterol total, HDL-colesterol, LDL-colesterol e VLDL-colesterol), renais (ureia, creatinina e ácido úrico) e glicêmicos foram medidos no soro dos indivíduos hipertensos e normotensos por meio de espectrofotometria UV/Vis, com utilização de reagentes Cobas® Substrates (Roche), conforme as recomendações do fabricante, e processados em equipamento COBAS INTEGRA® 400 Plus (Roche). Os íons sódio e potássio foram medidos no soro usando um eletrodo íon-seletivo com reagentes LS Científica de acordo com as recomendações do fabricante e processados em equipamento AVL 9180 (Roche). Os valores de LDL-colesterol foram determinados com base no risco global atribuído a ambos os grupos e o colesterol não-HDL foi calculado através da fórmula: não-HDL = colesterol total − HDL. ^14^

### Genotipagem

Para conhecer as frequências genotípicas e alélicas da população, amostras de sangue total com EDTA foram coletadas e utilizadas para extração de DNA utilizando o PureLink Genomic DNA Mini Kit (Thermo Fisher Scientific). Foi utilizado o sistema TaqMan® SNP Genotyping Assays system (Thermo Fisher Scientific) para PCR em tempo real para analisar a variante *GNB3* (Gene ID: 2784) rs5443:C>T (C__2184734_10). A mistura reacional foi preparada com 5 μL de TaqMan ® Master Mix e 0,50 μL de reagente de trabalho (primer/probe), totalizando 5,5 μL de reagente. As amostras de DNA foram diluídas com água livre de nuclease fornecendo 10 ng/μL, e 5,5 µL de reagente pré-preparado e 4,5 µL de amostra diluída foram carregados na placa de reação óptica de 96 poços MicroAmp™, totalizando um volume de 10 µL por poço. Fita adesiva foi utilizada para selar a placa que foi centrifugada a 1000 rpm e processada no instrumento 7500 FAST de PCR em tempo real. O software 7500 v2.3 foi utilizado para analisar os dados de discriminação alélica (Applied Biosystems, Thermo Fisher Scientific).

### Análise de dados

Foi realizada análise exploratória dos dados, e foram obtidas medidas de frequência absoluta e relativa para dados categóricos. O teste de Shapiro-Wilk foi utilizado para a normalidade dos dados contínuos. Para variáveis contínuas paramétricas, os dados foram expressos como média e desvio padrão (DP), e os dados não paramétricos foram expressos como mediana e intervalo interquartil.

Inicialmente, para identificar as variáveis sociodemográficas, clínicas, laboratoriais e genéticas da população associadas à hipertensão, foi realizada regressão logística univariada comparando a frequência relativa das variáveis categóricas. Após selecionar as variáveis com p < 0,25 na análise de regressão logística univariada, foi realizada regressão logística múltipla com ajuste para etnia; subsequentemente, usando a técnica reversa, foram selecionadas apenas variáveis com valor de p < 0,05 para compor o modelo final. Para as variáveis contínuas paramétricas, a média entre os grupos foi comparada pelo teste t de Student não pareado, e para comparar a mediana das variáveis contínuas não paramétricas, foi utilizado o teste de Mann-Whitney, uma vez que os grupos são independentes. Foram excluídos das análises participantes em uso de hipolipemiantes, hipoglicemiantes e uricosúricos. Todas as análises foram realizadas no software STATA V.13.0, considerando p < 0,05 como significativo. O equilíbrio de Hardy-Weinberg foi verificado pelo teste qui-quadrado de Pearson, utilizando o software genAIEx 6.5.

## Resultados

No presente estudo, 155 pacientes (média de idade 60,7 ± DP 7,5 anos) foram classificados como hipertensos (casos) e 155 (média de idade 58,2 ± DP 11,9 anos) como normotensos (controle). As características sociodemográficas e clínicas, bem como as características laboratoriais da população estudada, de acordo com a análise univariada, são apresentadas nas Tabelas 1 e 2, respectivamente.

**Tabela 1:** – Análise univariada das características sociodemográficas e clínicas da população estudada

Fatores	Hipertensão (n=155)		Controle (n=155)		Total (n=310)		OR (IC 95%)	p

	Média ± DP, n (%) ou mediana (1o – 3o quartil)							
Idade	61 (55-66)		60 (49-65)		60 (54-66)		0,9 (0,9-1,0)	**0,03** ^**c**^
Etnia								
Branca	44	(28,3)	61	(39,3)	105	(33,8)	1,0	
Preta	63	(40,6)	54	(34,8)	117	(37,7)	1,5 (0,9-2,6)	0,12
Parda	48	(30,9)	40	(25,8)	88	(28,3)	1,7 (0,9-2,9)	0,07
**Escolaridade**								
EMC/ESI/ESC/POS	36	(23,2)	50	(32,2)	86	(27,7)	1,0	
EFC/EMI	24	(15,4)	43	(27,7)	67	(21,6)	0,8 (0,4-1,9)	0,74
SI/EFI	95	(61,2)	62	(40)	157	(50,6)	2,7 (1,1-4,8)	**<0,05** ^**b**^
**Renda familiar (salários)**								
≥ 3	14	(9,0)	34	(21,9)	48	(15,4)	1,0	
>1 e < 3	134	(86,4)	116	(74,8)	250	(80,6)	3,1 (1,5-6,0)	<0,05 ^b^
≤ 1	7	(4,5)	5	(3,2)	12	(3,8)	1,7 (0,8-3,6)	0,16
**Tabagismo**								
Não fumante	97	(62.5)	120	(77,4)	217	(70)	1,0	
Ex-fumante	38	(24.5)	10	(6,4)	48	(15,4)	0,2 (0,1-0,4)	**<0,05** ^**b**^
Fumante	20	(12.9)	25	(16,1)	45	(14,5)	0,9 (0,4-1,7)	0,77
**IMC**	29 ± 5,3		26 ± 4,8		28 ± 5,3		0,9 (0,8-0,9)	**<0,001** ^**a**^

^a^ valores de p do teste t de Student não pareado; ^b^ regressão logística univariada; ^c^ valores de p do teste de Mann-Whitney. DP: desvio padrão; EFC: ensino fundamental completo; EFI: ensino fundamental incompleto; EMC: ensino médio completo; EMI: ensino médio incompleto; ESC: ensino superior completo; ESI: ensino superior incompleto; IC: intervalo de confiança; IMC: índice de massa corporal; OR: odds ratio; POS: pós-graduação; SI: sem instrução.

Fonte: dados compilados pelos autores do artigo.

**Tabela 2:** – Análise univariada das características bioquímicas da população

Fatores	Hipertensão (n=155)		Controle (n=155)		Total (n=310)		OR (IC 95%)	p

	n (%)							
Triglicerídeos								
Normais	86	(55,5)	119	(76,8)	205	(66,1)	1,0	
Alterados	69	(44,5)	36	(23,2)	105	(33,9)	2,7 (1,7-4,5)	<0,05
**Colesterol total**								
Normal	79	(51)	52	(33,5)	131	(42,2)	1,0	
Alterado	76	(49)	103	(66,4)	179	(57,7)	2,0 (0,3- 0,8)	<0,05
**HDL-colesterol**								
Normal	115	(74,2)	138	(89)	253	(81,6)	1,0	
Alterado	40	(25,8)	17	(10,9)	57	(18,4)	2,6 (1,4-4,8)	<0,05
**LDL-colesterol**								
Normal	26	(16,7)	4	(2,6)	30	(9,7)	1,0	
Alterado	129	(83,2)	151	(97,4)	280	(90,3)	7,6 (0,0-0,4)	<0,05
**Colesterol não-HDL**								
Normal	27	(17,4)	11	(7,1)	38	(12,2)	1,0	
Alterado	128	(82,6)	144	(92,9)	272	(87,7)	2,8 (0,2-0,7)	<0,05
**Ureia**								
Normal	141	(90,9)	152	(98,1)	293	(94,5)	1,0	
Alterada	14	(9)	3	(1,9)	17	(5,5)	4,0 (1,3-12,0)	<0,05
**Creatinina**								
Normal	144	(92,9)	154	(99,3)	298	(96,1)	1,0	
Alterada	11	(7,1)	1	(0,6)	12	(3,9)	11,8 (1,5-92,0)	<0,05
**Sódio**								
Normal	142	(91,6)	152	(98,1)	294	(94,8)	1,0	
Alterado	13	(8,4)	3	(1,9)	16	(5,2)	3,7(1,2-11,0)	<0,05
**Glicose**								
Normoglicemia	60	(38,7)	98	(63,2)	158	(51)	1,0	
Pré-diabetes	58	(37,4)	50	(32,2)	108	(34,8)	1,8 (1,1-3,1)	<0,05
Diabetes	37	(23,8)	7	(4,5)	44	(14,2)	7,5 (3,2-17,1)	<0,05
**Ácido úrico**								
Normal	90	(58,1)	122	(78,7)	212	(68,4)	1,0	
Alterado	65	(41,9)	33	(21,3)	98	(31,6)	2,7 (1,6-4,5)	<0,05

Todos os valores de p foram obtidos por meio de regressão logística multivariada. Valores de referência para parâmetros bioquímicos: triglicerídeos: até 150 mg/dL; colesterol total: até 190 mg/dL; HDL-colesterol: superior a 40 mg/dL; LDL-colesterol: inferior a 70 mg/dL; colesterol não-HDL: inferior a 100 mg/dL; ureia: até 50 mg/dL; creatinina: 0,4 a 1,4 mg/dL; sódio: 138 a 146 mEq/L; normoglicemia: inferior a 100 mg/dL, pré-diabetes: de 100 a 125 mg/dL, diabetes: superior ou igual a 126 mg/dL; ácido úrico: 2,4 a 5,7 mg/dL para mulheres e 3,4 a 7,0 mg/dL para homens. IC: intervalo de confiança; OR: odds ratio.

Fonte: dados compilados pelos autores do artigo.

As características sociais, clínicas e laboratoriais da população estudada de acordo com a análise multivariada realizada no modelo final são apresentadas na Tabela 3 .

**Tabela 3:** – Análise multivariada de fatores socioeconômicos, clínicos e bioquímicos da população

Fatores	Hipertensão (n=155)		Controle (n=155)		Total (n=310)		OR (IC 95%)	p

	Média ± DP, n (%) ou mediana (1o – 3o quartil)							
Idade	61 (55-66)		60 (49-65)		60 (54-66)		0,9 (0,8-0,9)	0,03
Escolaridade								
EMC/ESI/POS	36	(23,2)	50	(32,2)	86	(27,7)	1,0	
EFC/HIS	24	(15,4)	43	(27,7)	67	(21,6)	0,5 (0,2-1,5)	>0,05
SI/EFI	95	(61,2)	62	(40)	157	(50,6)	3,5 (1,9-6,5)	**<0,001**
**Tabagismo**								
Não fumante	97	(62,5)	120	(77,4)	217	(70)	1,0	
Ex-fumante	38	(24,5)	10	(6,4)	48	(15,4)	0,1 (0,1-0,9)	**<0,001**
Fumante	20	(12,9)	25	(16,1)	45	(14,5)	0,6 (0,2-1,3)	>0,05
**IMC**	29 ± 5,3		26 ± 4,8		28 ± 5,3		0,8 (0,8-0,9)	**<0,001**
**Triglicerídeos**								
Normais	86	(55,5)	119	(76,8)	205	(66,1)	1,0	
Alterados	69	(44,5)	36	(23,2)	105	(33,9)	0,5 (0,3-1,0)	0,04
**LDL-colesterol**								
Normal	26	(16,7)	4	(2,6)	30	(9,7)	1,0	
Alterado	129	(83,2)	151	(97,4)	280	(90,3)	5,9 (1,7-20,3)	**<0,001**
**Glicose**								
Normoglicemia	60	(38,7)	98	(63,2)	158	(51)	1,0	
Pré-diabetes	58	(37,4)	50	(32,2)	108	(34,8)	0,2 (0,1-0,3)	>0,05
Diabetes	37	(23,8)	7	(4,5)	44	(14,2)	0,6 (0,1-0,4)	**<0,001**
**Ácido úrico**								
Normal	90	(58,1)	122	(78,7)	212	(68,4)	1,0	
Alterado	65	(41,9)	33	(21,3)	98	(31,6)	0,5 (0,2-0,8)	**<0,001**

Todos os valores de p foram obtidos por meio de regressão logística multivariada. Valores de referência: triglicerídeos até 150mg/dL; LDL-colesterol menor do que 70 mg/dL; normoglicemia: menos do que 100 mg/dL, pré-diabetes: de 100 até 125 mg/dL, diabetes: maior ou igual a 126 mg/dL e ácido úrico: mulher 2,4 a 5,7 mg/dL, homem: 3,4 a 7,0 mg/dL. DP: desvio padrão; EFC: ensino fundamental completo; EFI: ensino fundamental incompleto; EMC: ensino médio completo; EMI: ensino médio incompleto; ESC: ensino superior completo; ESI: ensino superior incompleto; IC: intervalo de confiança; IMC: índice de massa corporal; OR: odds ratio; SI: sem instrução. Modelo ajustado por etnia.

Fonte: dados compilados pelos autores do artigo.

No presente estudo, no modelo final, foram encontradas diferenças significativas entre idade (p = 0,03), escolaridade (p < 0,001), tabagismo (p < 0,001), índice de massa corporal (p < 0,001), triglicerídeos (p = 0,04), LDL-colesterol (p < 0,001), glicose (p < 0,001) e ácido úrico (p < 0,001) quando comparados os grupos hipertensos e normotensos.

### Características alélicas e genotípicas da população estudada

A distribuição dos genótipos do gene *GNB3* analisados estava em equilíbrio de Hardy-Weinberg (p > 0,05).

Não foram encontradas diferenças significativas entre as frequências alélicas e genotípicas e as populações hipertensas e normotensas ( Tabela 4 ).

**Tabela 4:** – Frequência alélica e genotípica da variante GNB3 rs5443:C>T

Genótipos *GNB3*	Hipertenso (n=155)		Normotenso (n=155)		OR (IC 95%)	p

	n (%)					
rs5443:C>T						
Homozigoto CC	33	(21,3)	30	(19,3)	1,0	
Heterozigoto	79	(51)	79	(51)	0,9 (0,5-1,7)	0,901
Homozigoto TT	43	(27,7)	46	(29,7)	0,8 (0,4-1,6)	0,643
Alelo C	145	(46,8)	139	(44,8)	1,1 (0,8-1,5)	0,626
Alelo T	165	(53,2)	171	(55,2)	0,9 (0,7-1,3)	0,626

NOTA: Todos os valores de p foram obtidos por meio de regressão logística multivariada.

Fonte: dados compilados pelos autores do artigo.

### Análise do alelo T e características clínicas da população

**Figura 1:**
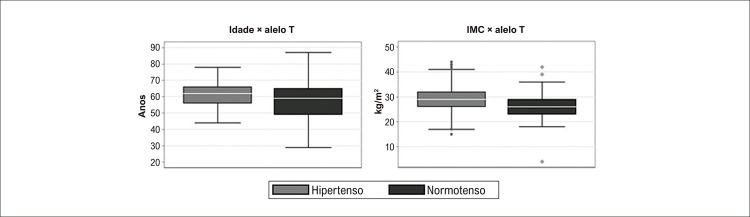
– Comparação entre (A) idade e (B) IMC de pacientes hipertensos e normotensos de acordo com a presença de pelo menos um alelo T. IMC: índice de massa corporal. Fonte: dados compilados pelos autores do artigos.

Na população estudada, os pacientes hipertensos que apresentavam pelo menos um alelo T eram significativamente mais velhos que os normotensos (p < 0,001) (Figura 1A). Da mesma forma, os pacientes hipertensos que possuíam pelo menos um alelo T apresentaram IMC médio significativamente maior do que aqueles que possuíam pelo menos um alelo T no grupo normotenso (p < 0,004) (Figure 1B).

### Análise de genótipos e dosagens bioquímicas

As Tabelas 5, 6 e 7 descrevem as características bioquímicas da população estudada em relação à presença do alelo T.

**Tabela 5:** – Perfil lipídico em relação ao alelo T

Fatores	Hipertenso (n=66)	Controle (n=112)	p

	Presença do alelo T		
Triglicerídeos, mediana (1 ^o^ – 3 ^o^ quartil)	127 (95,2-165)	104 (79,7-132,7)	**0,002 ^**a**^**
LDL-colesterol (média ± DP)	109,5 ± 43,6	130,2 ± 42,4	**0,003 ^**b**^**

^a^ valor de p do teste de Mann-Whitney; ^b^ valor de p do teste t de Student. Pacientes que faziam uso de agentes hipolipemiantes foram excluídos da análise. Valores de referência para parâmetros bioquímicos: triglicerídeos: até 150 mg/dL; LDL-colesterol: inferior a 70 mg/dL. DP: desvio padrão.

Fonte: dados compilados pelos autores do artigo.

**Tabela 6:** – Glicemia em relação ao alelo T

Fator	Hipertenso (n=74)	Controle (n=124)	p

	Presença do alelo T		
Glicose, mediana (1 ^o^ – 3 ^o^ quartil)	102,5 (93,2-118,7)	96 (89-104)	**0,004**

Valor de p do teste de Mann-Whitney. Pacientes em uso de agentes hipoglicemiantes foram excluídos da análise. Valores de referência: normoglicemia: inferior a 100 mg/dL; pré-diabetes: de 100 a 125 mg/dL; diabetes: maior ou igual a 126 mg/dL.

Fonte: dados compilados pelos autores do artigo.

**Tabela 7:** – Níveis de ácido úrico em relação ao alelo T

Fator	Hipertenso (n=121)	Controle (n=124)	p

	Presença do alelo T		
Ácido úrico (média ± DP)	5,9 ±1,6	5,3 ±1,3	**0,002**

Valor de p do teste t de Student não pareado. Pacientes em uso de medicamentos uricosúricos foram excluídos da análise. Valores de referência: ácido úrico: 2,4 a 5,7 mg/dL para mulheres e 3,4 a 7,0 mg/dL para homens. DP: desvio padrão.

Fonte: dados compilados pelos autores do artigo.

Nos pacientes hipertensos, os níveis de triglicerídeos, glicose e ácido úrico foram maiores naqueles que possuíam pelo menos um alelo T em comparação aos pacientes normotensos (p < 0,002, p < 0,004 e p = 0,002, respectivamente). Em contrapartida, em pacientes normotensos, as concentrações de LDL-colesterol foram mais altas naqueles que possuíam pelo menos um alelo T quando comparados aos hipertensos (p = 0,003).

## Discussão

A hipertensão resulta da interação de fatores genéticos e ambientais. ^1 , 5^ Considerando que algumas variantes genéticas têm o potencial de contribuir para a suscetibilidade a determinadas doenças, ^13^ o presente estudo investigou a influência da variante *GNB3* rs5443:C>T em pacientes hipertensos e normotensos.

A variante *GNB3* rs5443:C>T tem sido implicada em um risco aumentado de desenvolvimento de hipertensão, embora os resultados sejam inconsistentes. ^6 , 15^ No presente estudo, a análise da frequência alélica e genotípica não mostrou associação com hipertensão arterial, diferindo dos estudos que relataram essa associação em populações caucasianas, leste-asiáticas, alemãs e australianas. ^5 , 7 , 15^ Por outro lado, estudo realizado em outra população brasileira também não relatou associação entre os genótipos do polimorfismo C825T do gene *GNB3* e a hipertensão arterial. ^16^ Isso pode ser devido à frequência desigual do alelo T entre diferentes etnias, ^7^ especialmente na população brasileira que é altamente mista.

No presente estudo, o alelo T foi correlacionado com a idade, mostrando que os pacientes hipertensos que possuem pelo menos um alelo T são geralmente mais velhos que os normotensos, corroborando outros estudos realizados na China ^12^ e na Região Sudeste do Brasil. ^13^ Apesar disso, os indivíduos normotensos que possuem pelo menos um alelo T podem ter maior probabilidade de desenvolver hipertensão do que os indivíduos que não possuem o alelo T. ^6^ Assim, é importante que jovens normotensos que possuem pelo menos um alelo T tenham consciência de que são um grupo suscetível à hipertensão arterial, necessitando de maiores cuidados a fim de evitar o aparecimento da doença quando forem mais velhos.

Em relação ao índice de massa corporal, também obtivemos resultados significativos mostrando que foi maior em pacientes hipertensos que possuem pelo menos um alelo T em comparação aos normotensos. Foram relatados resultados semelhantes em populações alemãs, chinesas e sul-africanas. ^17^ A presença de pelo menos um alelo T sugere que o polimorfismo C825T do *GNB3* , localizado na região codificadora do gene, resulta em uma proteína funcional que aumenta a expressão da proteína G favorecendo a lipólise induzida por catecolaminas e induzindo a obesidade. ^18^ Além disso, a hipertensão arterial em indivíduos obesos pode ser devida ao aumento do volume de líquido extracelular e ao aumento do fluxo sanguíneo para os tecidos e do retorno venoso, contribuindo ao débito cardíaco. ^19^ Em pessoas obesas, o fluxo sanguíneo é maior por causa do excesso de tecido adiposo, bem como do fluxo sanguíneo para vários outros órgãos que hipertrofiam em resposta ao trabalho excessivo de demandas metabólicas, enquanto consomem oxigênio dos tecidos. O excesso de gordura favorece a síntese de citocinas e espécies reativas de oxigênio, gerando inflamação. Esse processo contribui para o desenvolvimento da disfunção endotelial, enrijecendo a vasculatura, o que pode desencadear aterosclerose e hipertensão arterial. ^20^

Em relação às análises bioquímicas, nossos resultados mostraram que possuir pelo menos um alelo T foi associado a níveis mais elevados de triglicerídeos, glicose e ácido úrico em pacientes hipertensos, bem como níveis mais altos de LDL-colesterol em pacientes normotensos.

Feng et al., ^21^ estudando uma população da África do Sul, também demonstraram níveis mais elevados de triglicerídeos e glicose em indivíduos hipertensos com pelo menos um alelo T quando comparados a indivíduos normotensos. A presença de pelo menos um alelo T sugere que o polimorfismo C825T do *GNB3* resulta em uma proteína funcional que influencia a lipólise induzida por catecolaminas, elevando o perfil lipídico na corrente sanguínea. ^18^ Além disso, o aumento da expressão da proteína G interfere nos níveis de glicose através dos níveis lipídicos por meio do metabolismo, desencadeando uma redução da sensibilidade à insulina no tecido muscular. ^22^

Em relação ao ácido úrico, Bührmann et al. ^23^ encontraram maiores concentrações de ácido úrico em pacientes que apresentavam pelo menos um alelo T na população alemã. A presença de pelo menos um alelo T sugere que o aumento da expressão da proteína G desencadeia intensa reabsorção de sódio, favorecendo a hipertensão arterial. Devido ao comprometimento endotelial/renal causado pela hipertensão arterial, acredita-se que haja deficiência na excreção de ácido úrico, aumentando sua concentração plasmática.

Nosso achado de níveis mais elevados de colesterol LDL em indivíduos normotensos com pelo menos um alelo T pode ser devido ao fato de as amostras biológicas terem sido coletadas durante o período da pandemia de COVID-19, o que pode ter favorecido o aumento do consumo de alimentos ultraprocessados combinado com um estilo de vida sedentário motivado pelo isolamento social. ^24 , 25^ Foram encontrados resultados semelhantes por Siffert et al., ^7^ que verificaram níveis mais elevados de LDL-colesterol em uma população normotensa sem doença pré-existente, com pelo menos um alelo T.

O presente estudo apresenta algumas limitações, uma vez que a amostra utilizada não pode ser representativa da população brasileira, além do pequeno tamanho amostral.

## Conclusão

Na população estudada, a presença de pelo menos um alelo T da variante *GNB3* rs5443:C>T foi relacionada a pacientes hipertensos com média de idade de 60 anos. Além disso, foi associada a níveis mais elevados de índice de massa corporal em indivíduos hipertensos e pode ser determinante de alterações em parâmetros bioquímicos, como perfil lipídico, glicemia e ácido úrico em pacientes hipertensos. Esses resultados mostram a importância do diagnóstico genético para prevenir as causas e consequências da doença, mesmo em uma população altamente miscigenada como a brasileira, e sugerem que a variante *GNB3* rs5443:C>T pode ser usada como uma alternativa fácil e de baixo custo e como um marcador genético precoce de alterações bioquímicas no processo hipertensivo. Para melhor compreender a influência da variante rs5443:C>T na alteração do perfil bioquímico de pacientes hipertensos, é essencial que novos estudos epidemiológicos sejam realizados em outras populações maiores e geneticamente distintas.

### Declaração de disponibilidade de dados

Os dados que suportam os resultados deste estudo estão disponíveis mediante solicitação à autora correspondente/ao autor correspondente. Os dados não estão disponíveis publicamente devido a restrições éticas ou de privacidade.
