# Supplementary material for: Variante da Subunidade Beta 3 da Proteína G ( GNB3 ) Está Associada a Alterações Bioquímicas em Pacientes Brasileiros com Hipertensão
Source: Arq Bras Cardiol. 2023 Dec 7;120(12):e20230396. [Article in Portuguese] doi: 10.36660/abc.20230396 (PMC10789371; doi:10.36660/abc.20230396)
Supplement: Translation

# Translation: G Protein Subunit Beta 3 (GNB3) Variant Is Associated with Biochemical Changes in Brazilian Patients with Hypertension

**Authors:** Lívia da Cunha Agostini, Nayara Nascimento Toledo Silva, Ana Cláudia Faria Lopes, André Sacramento Melo, Luciana Soares Moreira Bicalho, Tamires Cunha Almeida, Vanessa de Almeida Belo, Wendel Coura-Vital, Luiz Fernando de Medeiros Teixeira, Angélica Alves Lima, Glenda Nicioli da Silva

**Affiliations:** 1 Brasil Universidade Federal de Ouro Preto (UFOP), Ouro Preto, MG – Brasil; 2 Brasil Universidade Federal de Ouro Preto (UFOP) – Departamento de Análises Clínicas (DEACL), Ouro Preto, MG – Brasil; 3 Brasil Butantan Institute, São Paulo, SP – Brasil; 4 Brasil Universidade Federal de Ouro Preto – Programa de Pós-Graduação em Ciências Farmacêuticas (CiPharma) – Departamento de Farmácia (DEFAR), Ouro Preto, MG – Brasil; 5 Brasil Universidade Federal de Ouro Preto – Programa de Pós-Graduação em Ciências Farmacêuticas (CiPharma) – Departamento de Análises Clínicas (DEACL), Ouro Preto, MG – Brasil

**Keywords:** Alleles, Biochemical Reactions, Genotype, Hypertension

## Abstract

**Background:**

Genes and their variants associated with environmental factors contribute to the development of the hypertensive phenotype. The G protein beta 3 subunit gene (GNB3) is involved in the intracellular signaling process, and its variants have been related to susceptibility to arterial hypertension.

**Objective:**

To determine the association of the GNB3 variant (rs5443:C>T) with arterial hypertension, biochemical parameters, age, and obesity in hypertensive and normotensive individuals from Ouro Preto, Minas Gerais, Brazil.

**Method:**

The identification of variants was performed by real-time PCR, using the TaqMan® system, in 310 samples (155 hypertensive and 155 normotensive). Biochemical analyses (renal function, lipid profile and glycemia) were performed from the serum using UV/Vis spectrophotometry and ion-selective electrode. A multiple logistic regression model was used to identify factors associated with arterial hypertension. The analysis of continuous variables with normal distribution was performed using the unpaired Student’s t test; non-normal data were analyzed using Mann-Whitney. P < 0.05 was considered significant.

**Results:**

The rs5443:C>T variant was not associated with arterial hypertension in the evaluated population (p = 0.88). Regarding biochemical measures, the T allele was associated with high levels of triglycerides, glucose and uric acid in hypertensive individuals (p < 0.05).

**Conclusion:**

These results show the importance of genetic diagnosis to prevent the causes and consequences of diseases and imply that the GNB3 rs5443:C>T variant may be associated with changes in the biochemical profile in hypertensive individuals.

## Introduction

Arterial hypertension (AH) is a chronic disease responsible for several diseases often associated with metabolic disorders. It affects several target organs are and can lead to sudden death, stroke, peripheral arterial disease, heart failure, acute myocardial infarction, and chronic kidney disease. ^1 , 2^

The physiological regulation of blood pressure and the pathophysiological changes that lead to AH have a genetic component. It is assumed that 30% to 50% of the interindividual variability of blood pressure can be genetically stipulated. ^3 , 4^ The variant *GNB3* rs5443:C>T, located on chromosome 12p13, in the exon 10 region, has been reported to be associated with AH. ^3 , 5^

G proteins are part of a superfamily of proteins and are initially in an inactive state bound to intracellular receptors. When activated, they trigger amplifying enzymes and excite ion channels, performing signal transduction. ^6^ The variant *GNB3* rs5443:C>T is responsible for the exchange of the C allele for T, generating an alternative splicing of exon 9, eliminating 41 amino acids (498-620) from the protein, generating the truncated functional variant G3-s that exacerbates the G protein. ^6 , 7^ That triggers intracellular signaling managing the availability of sodium and potassium. In a hyperactive state, G protein increases sodium and water retention, contributing to the development of AH. ^7^

Some studies have investigated the association between the variant *GNB3* rs5443:C>T and blood pressure in other populations, and the results are controversial. ^5 - 8^ However, Chen et al. ^4^ discussed how variant *GNB3* rs5443:C>T can serve as early genetic marker of blood pressure salt sensitivity. The presence of the polymorphism generates a functional protein that triggers lipolysis through catecholamines, changing the lipid profile in the bloodstream. ^9^ Moreover, it causes a reduction in insulin sensitivity in muscle tissue and intense sodium reabsorption, favoring AH. ^10^ Due to the endothelial/renal impairment caused by AH, there is a deficiency in the excretion of some substances, such as urea, creatinine, and uric acid, increasing their plasma concentrations. ^1 , 9^

Taking into account the global population, the frequency of the C allele is 67%, and that of the T allele is 33%. Ethnic groups such as European, African, African-American, Asian, and Latin American have C and T allele frequencies around 69% and 31%; 28% and 72%; 28% and 72%; 46% and 54%; 54% and 46%, respectively. ^11^ Studies have shown different frequencies in different Brazilian populations. ^12 , 13^

Considering the importance of genetic variability in AH, this study aimed to determine whether the *GNB3* rs5443:C>T variant was associated with AH and whether it influenced kidney function, lipid profile, and blood glucose in a sample of Brazilian hypertensive and normotensive patients.

## Methods

### Ethical statement

The study was carried out in accordance with the criteria adopted by the University Ethics Committee (CAAE 22455119.0.0000.5150), in accordance with resolution 466/2012.

### Study design

The case-control study was carried out in 2021 in the city of Ouro Preto, Minas Gerais, Brazil. Individuals present in the Laboratory of Clinical Analysis of the Faculty of Pharmacy of the Federal University of Ouro Preto for biochemical tests were invited to participate of the study. To those who accepted, a questionnaire was applied in the smartphone application KoBoToolbox in order to obtain information on sociodemographic and behavioral data and medical history. Anthropometric measurements such as weight, height, and waist circumference were obtained using a bioimpedance scale, stadiometer, and measuring tape, respectively. Subsequently, blood samples were collected for biochemical and molecular evaluation.

After analyzing the questionnaire/medical record, the individuals were separated into two groups. Those who used medication for hypertension and had a previous diagnosis of the disease in their medical records were classified as hypertensive. Individuals who did not use antihypertensive medication and did not have a diagnosis of hypertension in their medical records were classified as controls (normotensive).

The sample number was defined to reach the 95% significance level that is crucial for genetic studies. Thus, the sample size was defined using the OpenEpi program, version 3.01, with a bilateral confidence level (1-alpha) of 95, power of 80%, ratio of controls to cases of 1, hypothetical proportion of controls with exposure of 33% of 8, and odds ratio of 2. With this, the sample size was estimated at approximately 138 patients for the control and case groups totaling 276 patients, according to the Kelsey test. In the end, the hypertensive group had 155 patients, 87 women and 68 men with a mean age of 60.7 years, and the control group also had 155 patients, 85 women and 70 men with a mean age of 58.2 years.

### Biochemical dosages

For the biochemical analyses, the participants fasted for 8 hours. Lipid profile (triglycerides, total cholesterol, HDL-cholesterol, LDL-cholesterol, and VLDL-cholesterol), renal (urea, creatinine, and uric acid), and blood glucose profile were measured in the serum of hypertensive and normotensive individuals by means of UV/Vis spectrophotometry, with the use of Cobas® Substrates (Roche) reagents according to the manufacturer’s recommendations and processed in COBAS INTEGRA® 400 Plus equipment (Roche). Sodium and potassium ions were measured in serum using an ion-selective electrode using LS Científica reagents, in accordance with the manufacturer’s recommendations, and processed in AVL 9180 equipment (Roche). LDL-cholesterol values were determined based on the overall risk attributed to both groups and non-HDL cholesterol was calculated using the formula: non-HDL = total cholesterol − HDL. ^14^

### Genotyping

To know the genotypic and allele frequencies of the population, EDTA whole blood samples were collected and used to extract DNA using the PureLink Genomic DNA Mini Kit (Thermo Fisher Scientific). The TaqMan® SNP Genotyping Assays system (Thermo Fisher Scientific) was used for real-time PCR to analyze the *GNB3* variant (Gene ID: 2784) rs5443:C>T (C__2184734_10). The reaction mixture was prepared with 5 μL of TaqMan ® Master Mix and 0.50 μL of working reagent (primer/probe), totaling 5.5 μL of reagent. DNA samples were diluted with nuclease-free water providing 10 ng/μL, and 5.5 µL of pre-prepared reagent and 4.5 µL of diluted sample were loaded into the MicroAmp™ 96-well optical reaction plate, totaling a volume of 10 µL per well. Adhesive tape was used to seal the plate and it was centrifuged at 1000 rpm and processed on the 7500 FAST real-time PCR instrument. The 7500 v2.3 software was used to analyze the allelic discrimination data (Applied Biosystems, Thermo Fisher Scientific).

### Data analysis

An exploratory data analysis was performed, and absolute and relative frequency measurements were obtained for categorical data. The Shapiro-Wilk test was used to test the normality of continuous data. For parametric continuous variables, data were expressed as mean and standard deviation (SD), and non-parametric data were expressed as median and interquartile range.

Initially, to identify the population’s sociodemographic, clinical, laboratory, and genetic variables associated with hypertension, univariate logistic regression was performed comparing the relative frequency of categorical variables. After selecting variables with p < 0.25 in the univariate logistic regression analysis, multiple logistic regression was performed with adjustment for ethnicity; subsequently, using the reverse technique, only variables with p value < 0.05 were selected to compose the final model. For parametric continuous variables, the mean between groups was compared using the unpaired Student’s t test, and to compare the median of non-parametric continuous variables, the Mann-Whitney test was used, since the groups are independent. Participants using lipid-lowering, hypoglycemic, and uricosuric drugs were excluded from the analyses. All analyses were performed using STATA V.13.0 software, considering p < 0.05 as significant. Hardy-Weinberg equilibrium was verified by Pearson’s chi-square test, using genAIEx 6.5 software.

## Results

In the present study, 155 subjects (mean age 60.7 ± SD 7.5 years) were classified as hypertensive (case), and 155 (mean age 58.2 ± SD 11.9 years) were classified as normotensive (control). The sociodemographic and clinical characteristics, as well as the laboratory characteristics of the population studied, according to the univariate analysis, are presented in Tables 1 and 2, respectively.

**Table 1:** – Univariate analysis of the sociodemographic and clinical characteristics of the study population

Factors	Hypertension (n=155)		Control (n=155)		Total (n=310)		OR (95% CI)	p

	Mean ± SD, n (%) or median (1st - 3rd quartiles)							
Age	61 (55-66)		60 (49-65)		60 (54-66)		0.9 (0.9-1.0)	**0.03** ^**c**^
Ethnicity								
White	44	(28.3)	61	(39.3)	105	(33.8)	1.0	
Black	63	(40.6)	54	(34.8)	117	(37.7)	1.5 (0.9-2.6)	0.12
Brown	48	(30.9)	40	(25.8)	88	(28.3)	1.7 (0.9-2.9)	0.07
**Schooling**								
CHS/IHE/CHE/POS	36	(23.2)	50	(32.2)	86	(27.7)	1.0	
CPS/IHS	24	(15.4)	43	(27.7)	67	(21.6)	0.8 (0.4-1.9)	0.74
WI/IPS	95	(61.2)	62	(40)	157	(50.6)	2.7 (1.1-4.8)	**<0.05** ^**b**^
**Family income (wages)**								
≥ 3	14	(9.0)	34	(21.9)	48	(15.4)	1.0	
>1 and < 3	134	(86.4)	116	(74.8)	250	(80.6)	3.1 (1.5-6.0)	<0.05 ^b^
≤ 1	7	(4.5)	5	(3.2)	12	(3.8)	1.7 (0.8-3.6)	0.16
**Smoking**								
Non-smoking	97	(62.5)	120	(77.4)	217	(70)	1.0	
Ex-smoker	38	(24.5)	10	(6.4)	48	(15.4)	0.2 (0.1-0.4)	**<0.05** ^**b**^
Smoker	20	(12.9)	25	(16.1)	45	(14.5)	0.9 (0.4-1.7)	0.77
**BMI**	29 ± 5.3		26 ± 4.8		28 ± 5.3		0.9 (0.8-0.9)	**<0.001** ^**a**^

^a^ unpaired Student’s t test p values; ^b^ univariate logistic regression; ^c^ Mann-Whitney test p values. BMI: body mass index; CHE: complete higher education; CHS: complete high school; CI: confidence interval; CPS: complete primary education; IHE: incomplete higher education; IHS: incomplete high school; IPS: incomplete primary education; OR: odds ratio; POS: postgraduate studies; SD: standard deviation; WI: without instruction.

Source: data compiled by the author.

**Table 2:** – Univariate analysis of the biochemical characteristics of the population

Factors	Hypertension (n=155)		Control (n=155)		Total (n=310)		OR (95% CI)	p

	n (%)							
Triglycerides								
Normal	86	(55.5)	119	(76.8)	205	(66.1)	1.0	
Changed	69	(44.5)	36	(23.2)	105	(33.9)	2.7 (1.7-4.5)	<0.05
**Total cholesterol**								
Normal	79	(51)	52	(33.5)	131	(42.2)	1.0	
Changed	76	(49)	103	(66.4)	179	(57.7)	2.0 (0.3- 0.8)	<0.05
**HDL-cholesterol**								
Normal	115	(74.2)	138	(89)	253	(81.6)	1.0	
Changed	40	(25.8)	17	(10.9)	57	(18.4)	2.6 (1.4-4.8)	<0.05
**LDL-cholesterol**								
Normal	26	(16.7)	4	(2.6)	30	(9.7)	1.0	
Changed	129	(83.2)	151	(97.4)	280	(90.3)	7.6 (0.0-0.4)	<0.05
**Non-HDL cholesterol**								
Normal	27	(17.4)	11	(7.1)	38	(12.2)	1.0	
Changed	128	(82.6)	144	(92.9)	272	(87.7)	2.8 (0.2-0.7)	<0.05
**Urea**								
Normal	141	(90.9)	152	(98.1)	293	(94.5)	1.0	
Changed	14	(9)	3	(1.9)	17	(5.5)	4.0 (1.3-12.0)	<0.05
**Creatinine**								
Normal	144	(92.9)	154	(99.3)	298	(96.1)	1.0	
Changed	11	(7.1)	1	(0.6)	12	(3.9)	11.8 (1.5-92.0)	<0.05
**Sodium**								
Normal	142	(91.6)	152	(98.1)	294	(94.8)	1.0	
Changed	13	(8.4)	3	(1.9)	16	(5.2)	3.7(1.2-11.0)	<0.05
**Glucose**								
Normoglycemia	60	(38.7)	98	(63.2)	158	(51)	1.0	
Prediabetes	58	(37.4)	50	(32.2)	108	(34.8)	1.8 (1.1-3.1)	<0.05
Diabetes	37	(23.8)	7	(4.5)	44	(14.2)	7.5 (3.2-17.1)	<0.05
**Uric acid**								
Normal	90	(58.1)	122	(78.7)	212	(68.4)	1.0	
Changed	65	(41.9)	33	(21.3)	98	(31.6)	2.7 (1.6-4.5)	<0.05

All p values were obtained using univariate logistic regression. Reference values for biochemical parameters: triglycerides: up to 150 mg/dL; total cholesterol: up to 190 mg/dL; HDL-cholesterol: greater than 40 mg/dL; LDL-cholesterol: lower than 70 mg/dL; non-HDL: less than 100 mg/dL; urea: up to 50 mg/dL; creatinine: 0.4 to 1.4 mg/dL; sodium: 138 to 146 mEq/L; normoglycemia: less than 100 mg/dL, prediabetes: from 100 to 125 mg/dL, diabetes: greater than or equal to 126 mg/dL; uric acid: 2.4 to 5.7 mg/dL for females and 3.4 to 7.0 mg/dL for males. CI: confidence interval; OR: odds ratio.

Source: data compiled by the author.

The social, clinical, and laboratory characteristics of the study population according to the multivariate analysis performed on the final model are shown in Table 3 .

**Table 3:** – Multivariate analysis of socioeconomic, clinical, and biochemical factors in the population

Factors	Hypertension (n=155)		Control (n=155)		Total (n=310)		OR (95% CI)	p

	Mean ± SD, n (%) or median (1st - 3rd quartiles)							
Age	61 (55-66)		60 (49-65)		60 (54-66)		0.9 (0.8-0.9)	0.03
Schooling								
CHS/IHE/POS	36	(23.2)	50	(32.2)	86	(27.7)	1.0	
CPS/HIS	24	(15.4)	43	(27.7)	67	(21.6)	0.5 (0.2-1.5)	>0.05
WI/IPS	95	(61.2)	62	(40)	157	(50.6)	3.5 (1.9-6.5)	**<0.001**
**Smoking**								
Non-smoking	97	(62.5)	120	(77.4)	217	(70)	1.0	
Ex-smoker	38	(24.5)	10	(6.4)	48	(15.4)	0.1 (0.1-0.9)	**<0.001**
Smoker	20	(12.9)	25	(16.1)	45	(14.5)	0.6 (0.2-1.3)	>0.05
**BMI**	29 ± 5.3		26 ± 4.8		28 ± 5.3		0.8 (0.8-0.9)	**<0.001**
**Triglycerides**								
Normal	86	(55.5)	119	(76.8)	205	(66.1)	1.0	
Changed	69	(44.5)	36	(23.2)	105	(33.9)	0.5 (0.3-1.0)	0.04
**LDL-cholesterol**								
Normal	26	(16.7)	4	(2.6)	30	(9.7)	1.0	
Changed	129	(83.2)	151	(97.4)	280	(90.3)	5.9 (1.7-20.3)	**<0.001**
**Glucose**								
Normoglycemia	60	(38.7)	98	(63.2)	158	(51)	1.0	
Prediabetes	58	(37.4)	50	(32.2)	108	(34.8)	0.2 (0.1-0.3)	>0.05
Diabetes	37	(23.8)	7	(4.5)	44	(14.2)	0.6 (0.1-0.4)	**<0.001**
**Uric acid**								
Normal	90	(58.1)	122	(78.7)	212	(68.4)	1.0	
Changed	65	(41.9)	33	(21.3)	98	(31.6)	0.5 (0.2-0.8)	**<0.001**

NOTE: All p values were obtained using multivariate logistic regression. Reference values: triglycerides up to 150mg/dL; LDL-cholesterol lower than 70 mg/dL; normoglycemia: less than 100 mg/dL, pre-diabetes: from 100 to 125 mg/dL, diabetes: greater than or equal to 126 mg/dL and uric acid: female 2.4 to 5.7 mg/dL, male: 3.4 to 7.0 mg/dL. BMI: body mass index; CHE: complete higher education; CHS: complete high school; CI: confidence interval; CPS: complete primary education; IHE: incomplete higher education; IHS: incomplete high school; IPS: incomplete primary education; OR: odds ratio; WI: without instruction. Model adjusted by ethnicity.

Source: data compiled by the author.

In the present study, in the final model, significant differences were found between age (p = 0.03), education (p < 0.001), smoking (p < 0.001), body mass index (BMI) (p < 0.001), triglycerides (p = 0.04), LDL-cholesterol (p < 0.001), glucose (p < 0.001), and uric acid (p < 0.001) when the hypertensive and normotensive groups were compared.

### Allelic and genotypic characteristics of the study population

The distribution of the *GNB3* gene genotypes analyzed were in Hardy-Weinberg equilibrium (p > 0.05).

No significant differences were found between the frequencies of alleles and genotypes and the hypertensive and normotensive populations ( Table 4 ).

**Table 4:** – Allelic and genotypic frequency of the variant GNB3 rs5443:C>T

Genotypes *GNB3*	Hypertensive (n=155)		Normotensive (n=155)		OR (95% CI)	p

	n (%)					
rs5443:C>T						
Homozygous CC	33	(21.3)	30	(19.3)	1.0	
Heterozygous	79	(51)	79	(51)	0.9 (0.5-1.7)	0.901
Homozygous TT	43	(27.7)	46	(29.7)	0.8 (0.4-1.6)	0.643
Allele C	145	(46.8)	139	(44.8)	1.1 (0.8-1.5)	0.626
Allele T	165	(53.2)	171	(55.2)	0.9 (0.7-1.3)	0.626

All p values were obtained using univariate logistic regression.

Source: data compiled by the author.

### Analysis of the T allele and clinical characteristics of the population

In the population studied, hypertensive individuals who had at least one T allele were significantly older than normotensive individuals (p < 0.001) (Figure 1A). Similarly, hypertensive subjects who carried at least one T allele had a significantly higher mean BMI than those who had at least one T allele in the normotensive group (p < 0.004) (Figure 1B).

**Figure 1:** – Comparison between (A) Age and (B) BMI of hypertensive and normotensive individuals according to the presence of at least one T allele. BMI: body mass index. Source: data compiled by the autor.

### Analysis of genotypes and biochemical dosages

Tables 5, 6, and 7 describe the biochemical characteristics of the study population in relation to the presence of the T allele.

**Table 5:** – Lipid profile in relation to the T allele

Factors	Hypertensive (n=66)	Control (n=112)	p

	Presence of T allele		
Triglycerides, median (1st – 3rd quartiles)	127 (95.2-165)	104 (79.7-132.7)	**0.002 ^**a**^**
LDL-cholesterol (mean ± SD)	109.5 ± 43.6	130.2 ± 42.4	**0.003 ^**b**^**

^a^ p value from the Mann-Whitney test; ^b^ p value from Student’s t test ^b^ . Patients who used lipid-lowering drugs were excluded from the analysis. Reference values for biochemical parameters: triglycerides: up to 150 mg/dL; LDL-cholesterol: lower than 70 mg/dL. SD: standard deviation.

Source: data compiled by the author.

**Table 6:** – Glycemia in relation to the T allele

Factor	Hypertensive (n=74)	Control (n=124)	p

	Presença do alelo T		
Glucose, median (1st – 3rd quartiles)	102.5 (93.2-118.7)	96 (89-104)	**0.004**

Mann-Whitney test p value. Patients using hypoglycemic agents were excluded from the analysis. Reference values: normoglycemia: less than 100 mg/dL; pre-diabetes: from 100 to 125 mg/dL; diabetes: greater than or equal to 126 mg/dL.

Source: data compiled by the author

**Table 7:** – Uric acid levels in relation to the T allele

Factor	Hypertensive (n=121)	Control (n=124)	p

	Presence of T allele		
Uric acid (mean ± SD)	5.9 ±1.6	5.3 ±1.3	**0.002**

Unpaired Student’s t test p value. Patients using uricosuric drugs were excluded from the analysis. Reference values: uric acid: 2.4 to 5.7 mg/dL for females and 3.4 to 7.0 mg/dL for males. SD: standard deviation.

Source: data compiled by the author.

In hypertensive patients, triglyceride, glucose, and uric acid levels were higher in those who had at least one T allele compared to normotensive patients (p < 0.002, p < 0.004, and p = 0.002, respectively). In contrast, in normotensive subjects, LDL-cholesterol concentrations were higher in those who had at least one T allele when compared to hypertensive subjects (p = 0.003).

## Discussion

Hypertension results from the interaction of genetic and environmental factors. ^1 , 5^ Considering that some genetic variants have the potential to contribute to susceptibility to certain diseases, ^13^ the present study examined the influence of the variant *GNB3* rs5443:C>T in hypertensive and normotensive individuals.

The *GNB3* rs5443:C>T variant has been implicated in an increased risk of developing hypertension, although results are inconsistent. ^6 , 15^ In this study, analysis of allele and genotypic frequency showed no association with AH, differing from studies that reported this association in Caucasian, East Asian, German, and Australian populations. ^5 , 7 , 15^ On the other hand, a study carried out in another Brazilian population also reported no association between the genotypes of the C825T polymorphism of the *GNB3* gene and AH. ^16^ This may be due to the uneven frequency of the T allele among different ethnicities, ^7^ especially in the highly mixed Brazilian population.

In this study, the T allele was correlated with age, showing that hypertensive individuals who have at least one T allele are generally older than normotensive individuals, corroborating other studies carried out in China ^12^ and in the Southeast Region of Brazil. ^13^ Despite that, normotensive individuals who have at least one T allele may be more likely to develop hypertension than individuals who do not have the T allele. ^6^ Thus, it is important that normotensive young people who have at least one T allele are aware that they are a susceptible group to AH, requiring greater care in order to avoid the onset of the disease when they are older.

Regarding BMI, we also obtained significant results showing that this index was higher in hypertensive individuals who have at least one T allele compared to normotensive individuals. Similar results have been reported in German, Chinese, and South African populations. ^17^ The presence of at least one T allele suggests that the C825T polymorphism of *GNB3* , located in the coding region of the gene, results in a functional protein that enhances the expression of the G protein favoring catecholamine-induced lipolysis and inducing obesity. ^18^ Furthermore, AH in obese individuals may be due to increased extracellular fluid volume and increased blood flow to tissues and venous return, contributing to cardiac output. ^19^ In obese people, blood flow is greater because of extra adipose tissue, as well as blood flow to several other organs that hypertrophy in response to excessive work of metabolic demands, while consuming tissue oxygen. Excess fat favors the synthesis of cytokines and oxygen-reactive species and generates inflammation. This process contributes to the development of endothelial dysfunction, stiffening the vasculature, which can trigger atherosclerosis and AH. ^20^

Regarding biochemical analyses, our results showed that having at least one T allele was associated with higher levels of triglycerides, glucose, and uric acid in hypertensive patients and LDL-cholesterol in normotensive patients. Feng et al., ^21^ studying a population from South Africa, also demonstrated higher levels of triglycerides and glucose in hypertensive individuals with at least one T allele when compared to normotensive individuals. The presence of at least one T allele suggests that the C825T polymorphism of *GNB3* results in a functional protein that influences catecholamine-induced lipolysis, elevating the lipid profile in the bloodstream. ^18^ In addition, increased expression of G protein interferes with glucose levels through lipid metabolism, triggering a reduction in insulin sensitivity in muscle tissue. ^22^

With respect to uric acid, Bührmann et al. ^23^ found higher uric acid concentrations in patients who had at least one T allele in the Germanic population. The presence of at least one T allele suggests that increased G protein expression triggers intense sodium reabsorption, favoring AH. Due to the endothelial/renal impairment caused by AH, it is believed that there is a deficiency in the excretion of uric acid, increasing its plasma concentration.

Our finding of higher levels of LDL-cholesterol in normotensive individuals with at least one T allele may be due to the fact that the biological samples were collected during the period of the COVID-19 pandemic, which may have favored increased ultra-processed food consumption combined with a sedentary lifestyle motivated by social isolation. ^24 , 25^ Similar results were found by Siffert et al. ^7^ who showed higher levels of LDL-cholesterol in a normotensive population without pre-existing disease, with at least one T allele.

The study has some limitations, since the sample used could not be representative of the Brazilian population, in addition to small sample size.

## Conclusion

In conclusion, in the population studied, the presence of at least one T allele of the *GNB3* rs5443:C>T variant was related to hypertensive patients with a mean age of 60 years. In addition, it was associated with higher BMI levels in hypertensive individuals and may be a determinant of changes in biochemical parameters, such as lipid profile, blood glucose, and uric acid in hypertensive individuals. These results show the importance of genetic diagnosis to prevent the causes and consequences of the disease, even in a highly mixed population such as the Brazilian one, and they suggest that the *GNB3* rs5443:C>T variant can be used as an easy, inexpensive, and early genetic marker of biochemical alterations in the hypertensive process. To better understand the influence of the rs5443:C>T variant on the alteration of the biochemical profile in hypertensive patients, it is essential that new epidemiological studies be carried out in other larger and genetically distinct populations.

### Data accessibility statement

The data that support the findings of this study are available on request from the corresponding author. The data are not publicly available due to privacy or ethical restrictions.
